# A miniature, subterranean, blind cobitid loach, *Gitchak nakana*, new genus and species, is the first groundwater-dwelling fish from Northeast India

**DOI:** 10.1038/s41598-026-40425-6

**Published:** 2026-02-26

**Authors:** Ralf Britz, Wimarithy K. Marak, Kangjam Velentina, Yumnam Lokeshwor, Rajeev Raghavan, Amanda K. Pinion, Lukas Rüber

**Affiliations:** 1 Senckenberg Naturhistorische Sammlungen Dresden, Dresden, Germany; 2 Assam Don Bosco University, Guwahati, Assam India; 3 Dhanamanjuri University, Imphal, Manipur India; 4 Department of Fisheries Resource Management, Kerala University of Fisheries and Ocean Studies (KUFOS), Kochi, India; 5 Naturhistorisches Museum Bern, Bern, Switzerland; 6 Aquatic Ecology and Evolution, Institute of Ecology and Evolution, University of Bern, Bern, Switzerland

**Keywords:** Ecology, Ecology, Zoology

## Abstract

Subterranean animals are commonly met with considerable fascination, by both laymen and biologists. While most of these animals have been reported from caves, some species have adapted to other underground habitats. One special subterranean aquatic habitat are aquifers, which are home to a number of invertebrates and fishes. Of the more than 300 known subterranean fishes, fewer than 10% have been recovered from aquifers and are encountered only rarely and serendipitously. Here we report the discovery of a tiny, blind loach of the family Cobitidae from a dug-out well in Assam, India, which exhibits a number of characters commonly associated with subterranean life, so-called troglomorphies. This loach, described here as *Gitchak nakana*, represents a new genus and species, differing from all other genera of cobitids by a number of unique characters. The most unusual among these is the complete lack of a skull roof with the brain covered dorsally only by skin. *Gitchak nakana* is the first aquifer-dwelling (phreatobitic) fish from Northeast India, and marks the first discovery of a previously unknown subterranean fauna in this part of Asia.

**Supplementary Information:**

The online version contains supplementary material available at 10.1038/s41598-026-40425-6.

## Introduction

The subterranean realm offers fascinating habitats which require considerable modifications from its inhabitants^1,2^. Since scientific description of the Olm, *Proteus anguinus*, in the 18th century^3^, subterranean animals with their often-otherworldly appearance have peaked the interest of researchers and the public alike. Animals adapted to subterranean life often share similar morphological features, termed troglomorphies, and these include reduction or absence of eyes and pigment and augmentation of non-visual senses frequently combined with an increase in the length of body appendages^2,4^. The most common subterranean habitat are caves which are present on all continents except Antarctica^5^. Caves that contain water bodies have regularly evolved a unique aquatic fauna, which includes various invertebrates, such as crustaceans, but also fishes^6,7^. The vast majority of the more than 300 fish taxa recorded from subterranean habitats are from caves^7^. In sharp contrast, surprisingly few fish species have been recovered from another subterranean aquatic habitat, groundwater aquifers, and these phreatobites^8^are usually rare and represented by only few specimens in museum collections^9^.

**Fig. 1:**
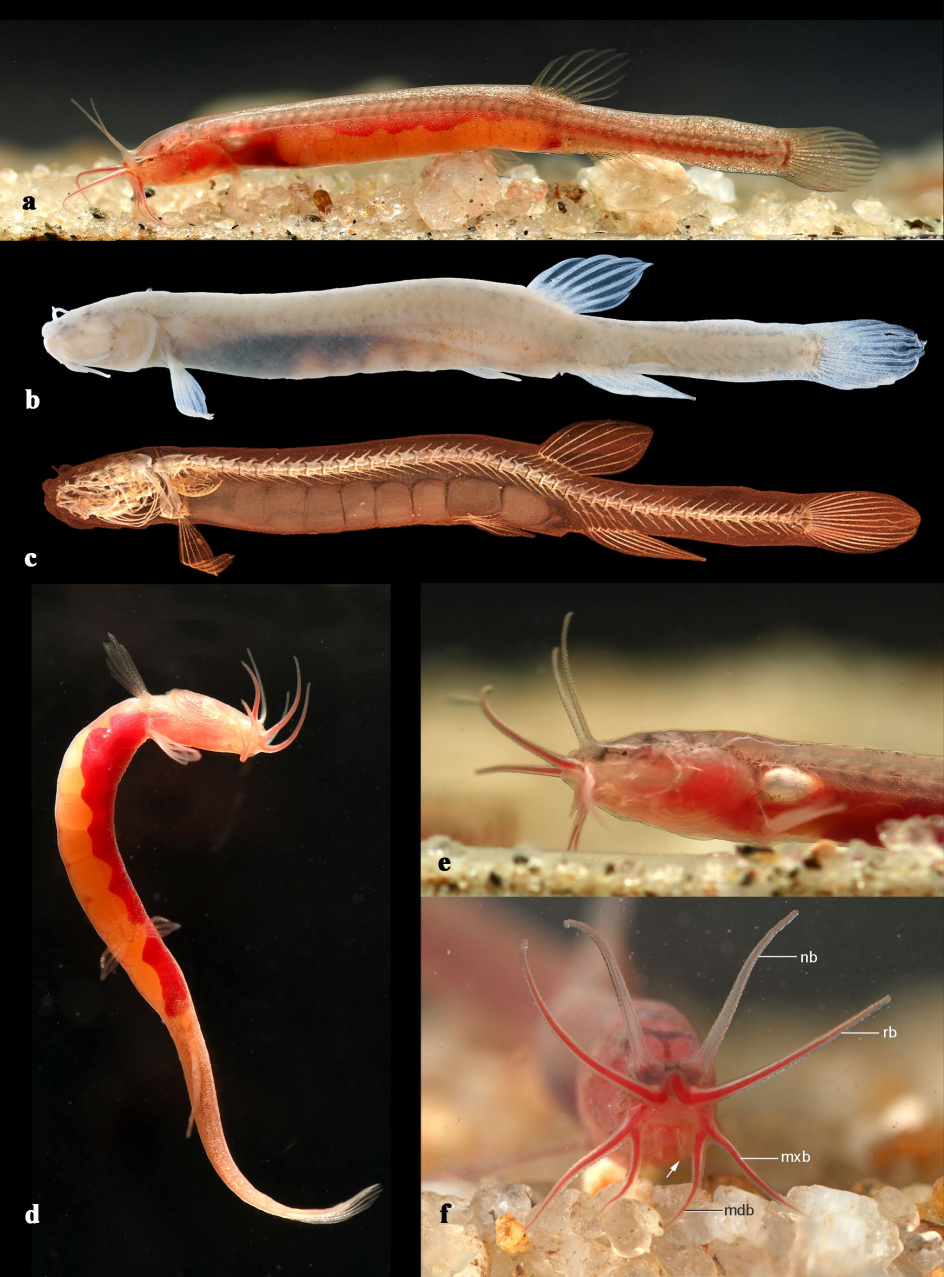
*Gitchak nakana*. (**a**) ZSI FF11123, holotype, 20.8 mm SL, in life, sides reversed, showing overall appearance, note presence of large eggs in body cavity and numerous fat globules (reflective spheres) along dorsal midline and postanal ventral midline. (**b**) same specimen, after preservation. (**c**) same specimen, µCT-image to illustrate presence of eight large eggs arranged in a longitudinal series. (**d**) same specimen, in life, actively swimming in the water column; note large yellow eggs and blood red liver. (**e**) KUFOS2025.F.11.51, non-type, 16.4 mm SL, in life, close-up of lateral head and body; note swimbladder visible through body wall. (**f**) KUFOS:2025.FT.11.6, paratype, 20.0 mm SL, frontal view of head to illustrate crown of barbels; note large-calibre red blood vessels supplying rostral (rb), maxillary (mxb) and mandibular (mdb) barbels and small-calibre vessels supplying nasal (nb) and tiny mental barbels (marked by arrow).

**Fig. 2:**
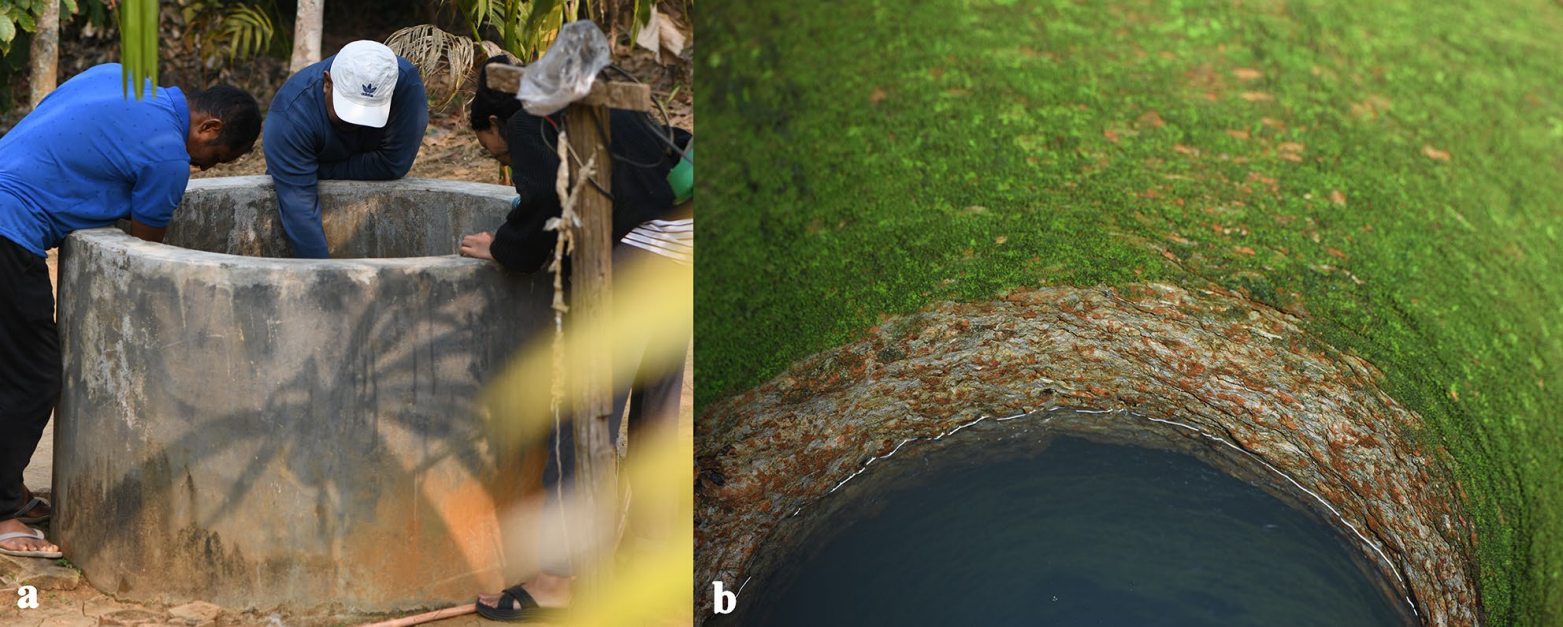
Well from which all specimens of *Gitchak nakana* were collected. (**a**) View from outside showing top formed by a ring of concrete. (**b**) View inside illustrating clear groundwater and internal walls.

Here, we report the discovery of such a phreatobitic fish, a subterranean troglomorphic loach from a dug-out open well in Assam, India (Figs. 1 and 2). This new genus and species of miniature, pigmentless and blind cobitid loach was collected on three occasions from the same well in a small village at the foothills of the Shillong Plateau close to the Brahmaputra valley in the west of Assam. Caves on the Shillong Plateau are home to several completely blind and pigmentless subterranean fishes, incl. two nemacheilid loaches (*Schistura papulifera* Kottelat, Harries & Proudlove 2007^10^; *Schistura larketensis* Choudhury et al. 2017^11^) and the world’s largest subterranean fish, *Neolissochilus pnar* Dahanukar et al. 2023^12^. The new cobitid loach, however, is the first phreatobitic fish species and the first subterranean cobitid from Northeast India. This paper serves to describe this new species, to provide details on its highly unusual skeletal anatomy and to offer a hypothesis of its phylogenetic position among cobitid loaches.

## Results

### Taxonomy *Gitchak*, new genus

urn: lsid: zoobank.org: act: B88B139A-ACA7-4B66-A89E-B95BC29E1798.

**Fig. 3:**
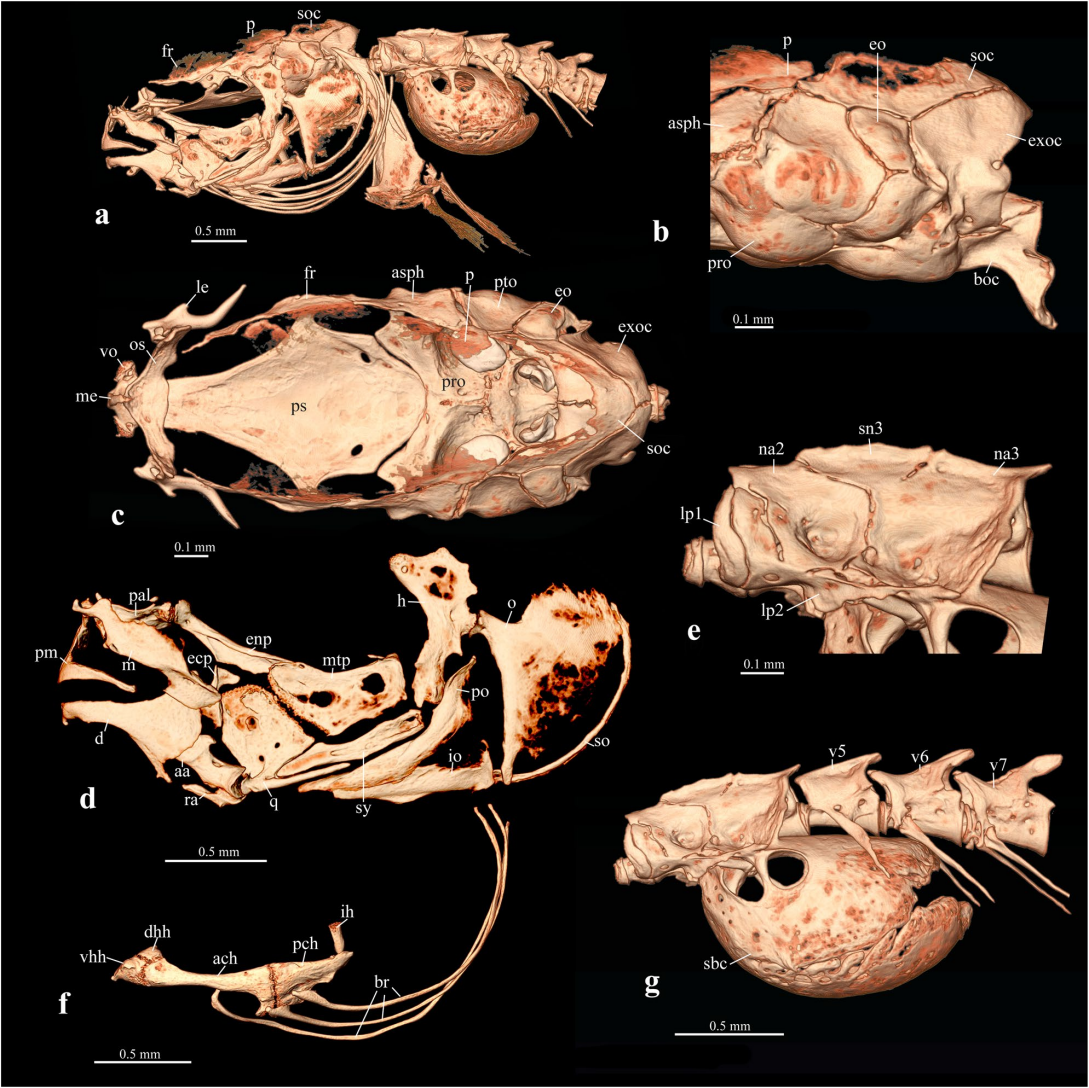
*Gitchak nakana*, ZSI FF11124, 20.2 mm SL. CT-images of head and anterior vertebral column in lateral view (**a**) and select complexes (**b**–**g**), illustrating unique and highly unusual characters. (**b**) Occipital part of skull in lateral view, note absence of foramen in exoccipital (exoc). (**c**) Neurocranium in dorsal view, note absence of skull roof and unique anterior position of orbitosphenoid (os) and suborbital spine. (**d**) Hyopalatine arch in lateral view, note absence of foramen between quadrate (q) and metapterygoid (mtp). (**e**) Weberian centra and neural arches in lateral view, note dorsally directed lateral process of first vertebra (lp1) completely covering scaphium laterally. (**f**) Hyoid, interhyal and branchiostegal rays, note very long dorsally curving branchistegal rays (br). (**g**) Weberian vertebrae and post-Weberian vertebrae 5–7 in lateral view, note long, ovoid swimbladder capsule (sbc) extending to anterior margin of seventh vertebra. Other abbreviations: ach: anterior ceratohyal; asph: autosphenotic; boc: basioccipital; d: dentary; dhh: dorsal hypohyal; ecp: ectopterygoid; enp: endopterygoid; eo: epiotic; h: hyomandibular; ih: interhyal; io: interopercle; le: lateral ethmoid; lp2: lateral process of vertebra 2; m: maxilla; me: mesethmoid; na2: neural arch of second vertebra; na3: neural arch of third vertebra; o: opercle; p: parietal; pch: posterior ceratohyal; pm: premaxilla; po: preopercle; pro: prootic; ps: parasphenoid; pto: pterotic; ra: retroarticular; sn3: supraneural 3; so: subopercle; soc: supraoccipital; sy: symplectic; V5,6,7: vertebra 5,6,7; vhh: ventral hypohyal; vo: vomer.

**Diagnosis.** *Gitchak* (Figs. 1 and 3) is a member of the family Cobitidae as evidenced by (i) the modification of lateral ethmoid into a bifurcated subocular spine, which articulates with the orbitosphenoid, (ii) the reduction of the endopterygoid into a rod-like element, (iii) the absence of contact between orbitosphenoid and pterosphenoid and (iv) the outer arm of the os suspensorium completely surrounding anterior swimbladder chamber^13,14^.

**Fig. 4:**
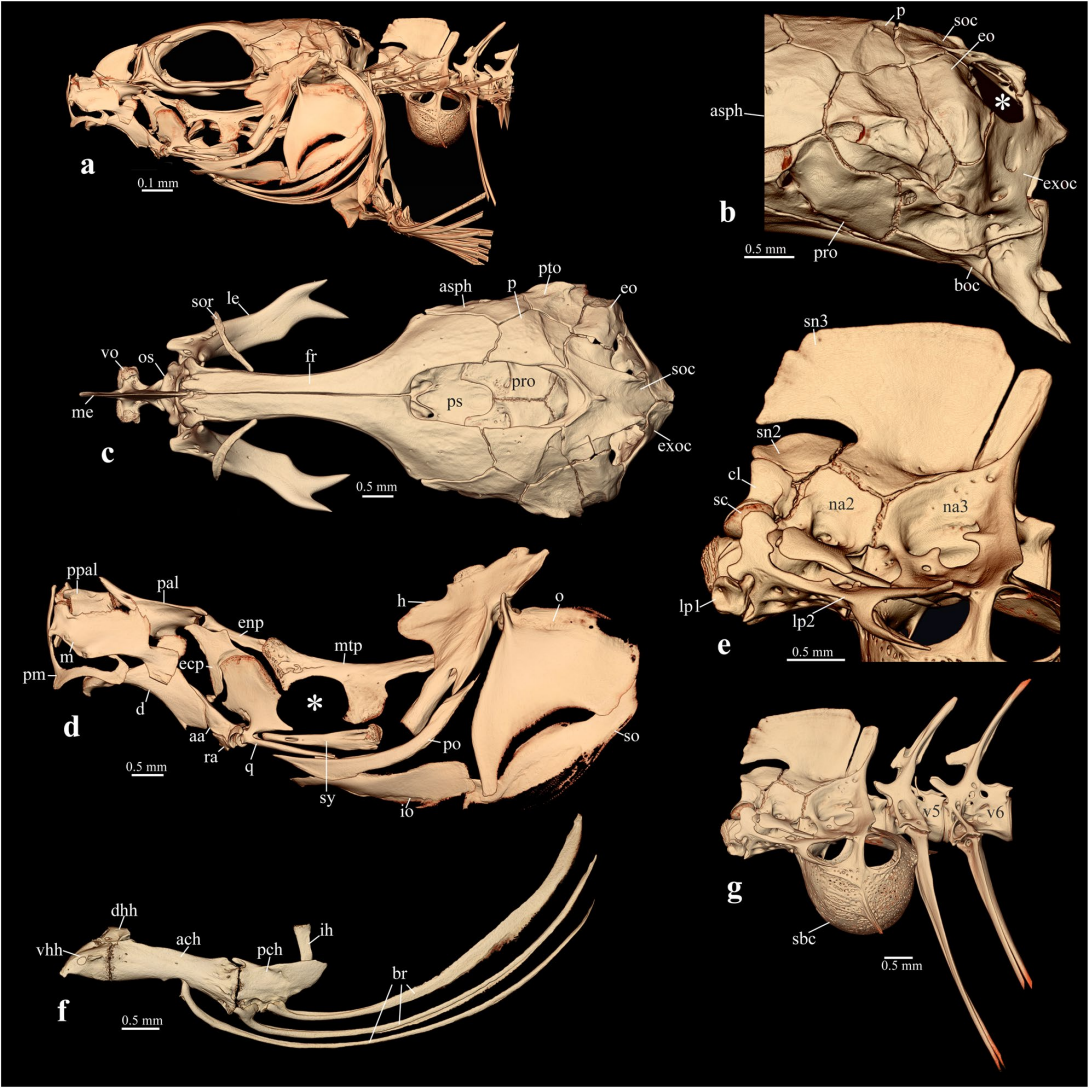
*Lepidocephalichthys guntea*, ADBU-MF 5597, 56.6 mm SL. µCT-images of head and anterior vertebral column in lateral view (middle) and select complexes for comparison with *Gitchak nakana* (Fig. 3): (**a**) lateral view of head. (**b**) Lateral view of occiput, note presence of exoccipital foramen (marked with *). (**c**) Dorsal view of skull, note complete skull roof. (**d**) Lateral view of hyopalatine arch, note quadrato-metapterygoid foramen (marked with *). (**e**) Lateral view of Weberian centra, first lateral process (lp1) short. (**f**) Lateral view of hyoid and branchiostegal rays (br). (**g**) Lateral view of Weberian and post-Weberian vertebrae, note small and short swimbladder capsule (sbc) only reaching posterior margin of vertebra 5. Other abbreviations: ach: anterior ceratohyal; asph: autosphenotic; boc: basioccipital; cl: claustrum; d: dentary; dhh: dorsal hypohyal; ecp: ectopterygoid; enp: endopterygoid; eo: epiotic; exoc: exoccipital; h: hyomandibular; ih: interhyal; io: interopercle; le: lateral ethmoid; lp2: lateral process of vertebra 2; m: maxilla; me: mesethmoid; mtp: metapterygoid; na2: neural arch of second vertebra; na3: neural arch of third vertebra; o: opercle; p: parietal; pal: autopalatine; ppal: preautopalatine; pch: posterior ceratohyal; pm: premaxilla; po: preopercle; pro: prootic; ps: parasphenoid; pto: pterotic; q: quadrate; ra: retroarticular; sc: scaphium; sn2: supraneural 2; sn3: supraneural 3; so: subopercle; sor: supraorbital; soc: supraoccipital; sy: symplectic; V5,6: vertebra 5,6; vhh: ventral hypohyal; vo: vomer.

**Fig. 5:**
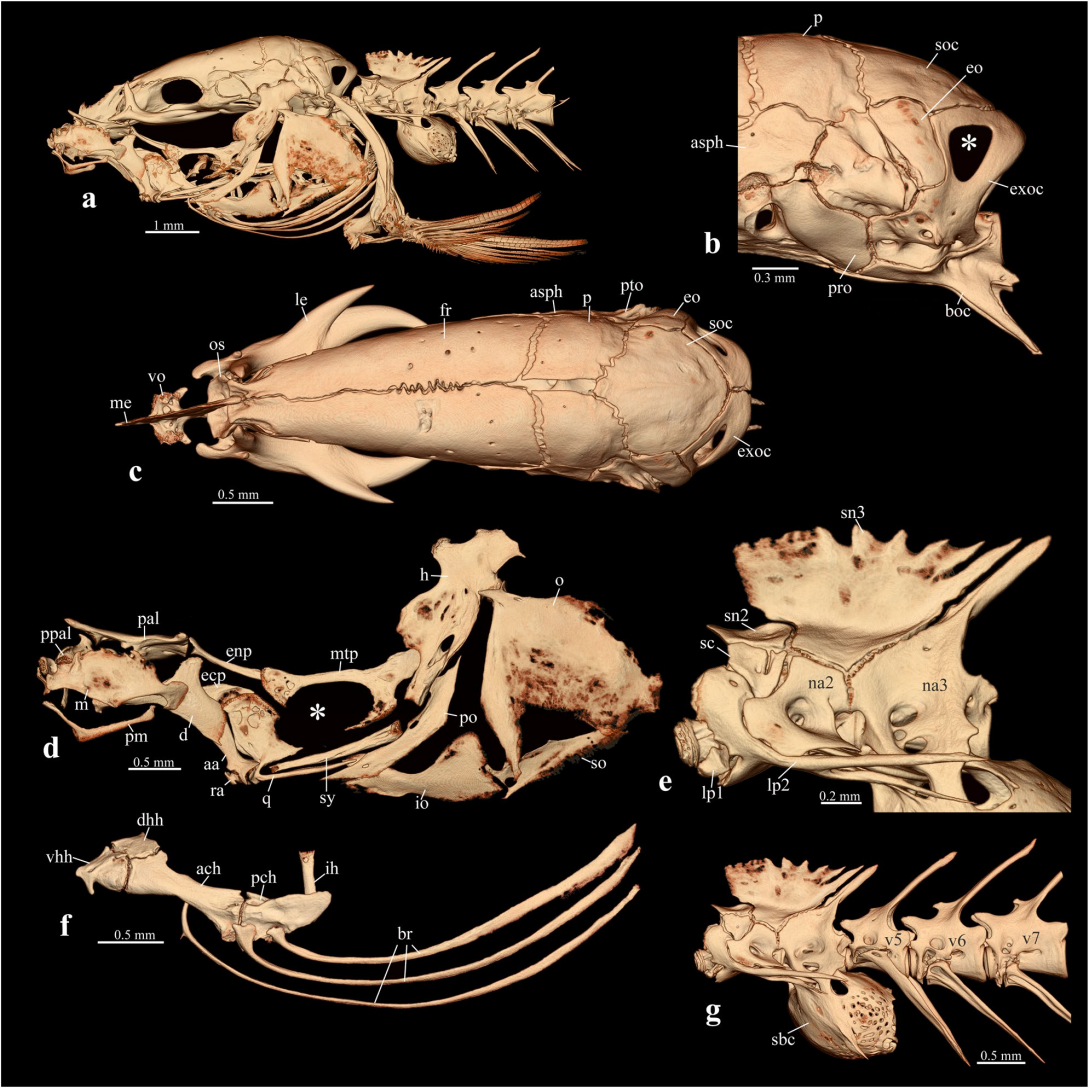
*Pangio pangia*, ADBU-MF 5282, 41.8 mm SL. µCT-images of head and anterior vertebral column in lateral view (middle) and select complexes for comparison with *Gitchak nakana* (Fig. 3): (**a**) lateral view of head. (**b**) Lateral view of occiput, note presence of exoccipital foramen (marked with *). (**c**) Dorsal view of skull, note complete skull roof. (**d**) Lateral view of hyopalatine arch, note quadrato-metapterygoid foramen (marked with *). (**e**) Lateral view of Weberian centra, first lateral process (lp1) short. (**f**) lateral view of hyoid and branchiostegal rays (br). (**g**) Lateral view of Weberian and post-Weberian vertebrae, note small and short swimbladder capsule (sbc) only reaching posterior margin of vertebra 5. ach: anterior ceratohyal; asph: autosphenotic; boc: basioccipital; cl: claustrum; d: dentary; dhh: dorsal hypohyal; ecp: ectopterygoid; enp: endopterygoid; eo: epiotic; h: hyomandibular; ih: interhyal; io: interopercle; le: lateral ethmoid; lp2: lateral process of vertebra 2; m: maxilla; me: mesethmoid; mtp: metapterygoid; na2: neural arch of second vertebra; na3: neural arch of third vertebra; o: opercle; p: parietal; pch: posterior ceratohyal; pm: premaxilla; po: preopercle; pal: autopalatine; ppal: preautopalatine; pro: prootic; ps: parasphenoid; pto: pterotic; q: quadrate; ra: retroarticular; sc: scaphium; sn2: supraneural 2; sn3: supraneural 3; so: subopercle; soc: supraoccipital; sy: symplectic; V5,6,7: vertebra 5,6,7; vhh: ventral hypohyal; vo: vomer.

**Fig. 6:**
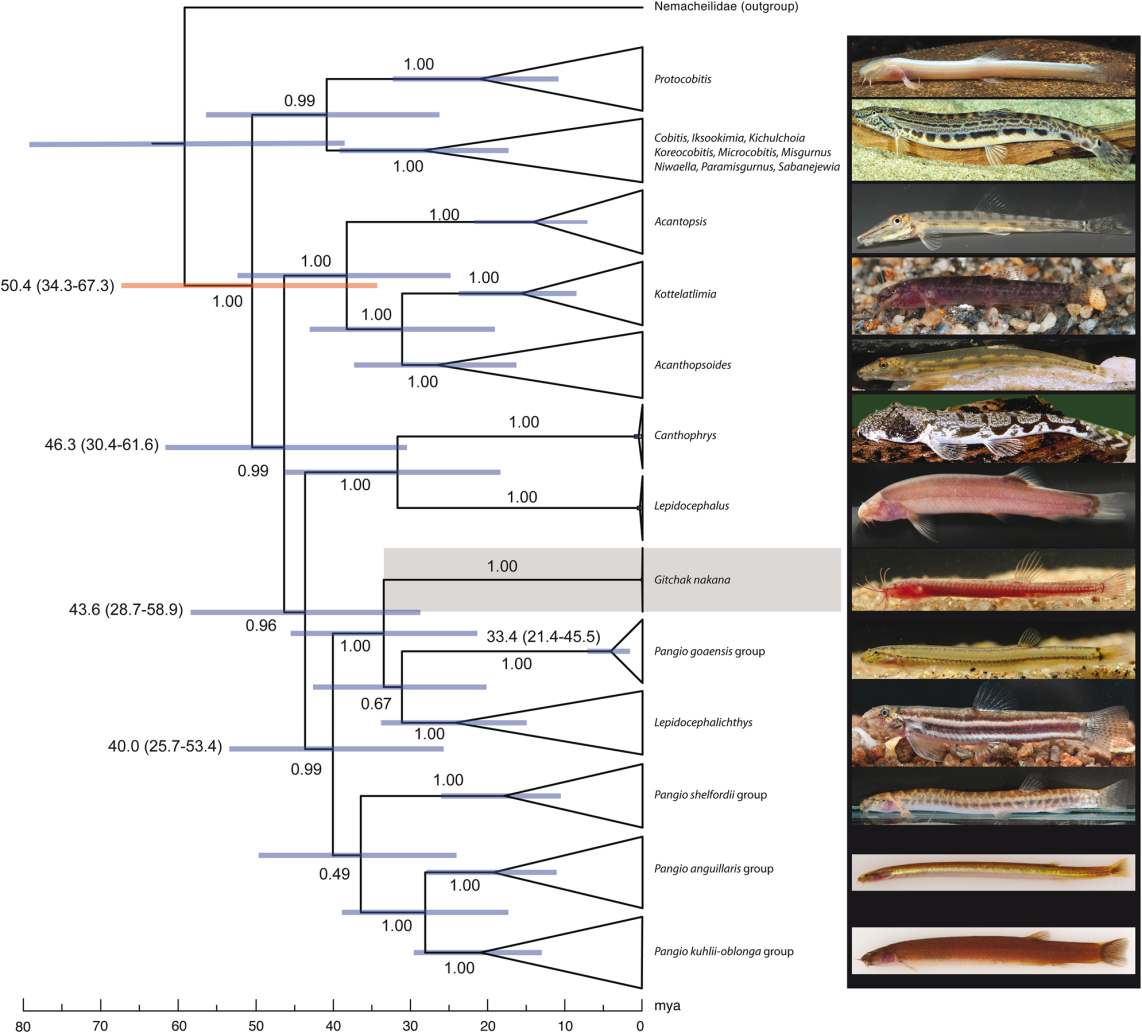
Molecular time tree from the BEAST analysis of combined cytb and RAG1 data set of Cobitidae with major clades collapsed to investigate phylogenetic position and minimum age of *Gitchak* (highlighted in grey). The secondary calibration interval obtained from the literature used to calibrate the tree is highlighted in red and age estimates and their confidence intervals are given for select nodes with their posterior probability support values shown above or below branches. The uncollapsed time tree is shown in Supplementary Fig. 1. Photos with credit from top to bottom: *Protocobitis longibarba* (Qin et al. 2025; CC BY 4.0); *Cobitis elongatoides*, A. Hartl; *Acantopsis* cf. *choirorhynchos*, H.H.Tan; *Kottelatlimia katik*, L. Rüber; *Acanthopsoides robertsi*, *Canthophrys gongota* F. Schäfer; *Lepidocephalus spectrum*, H.H. Tan; *Gitchak nakana*, *Pangio goaensis*, *Lepidocephalichthys guntea*, R. Britz; *P. shelfordii*, *P. anguillaris*, *Pangio oblonga*, H.H. Tan.

*Gitchak* is easily distinguished from all other genera of cobitids by (1) the absence of a skull roof, with the frontal and parietal being restricted to the side of the skull and failing to meet their antimeres in the dorsal midline (vs. presence of skull roof) [compare Figs. 3c, 4c and 5c]; (2) absence of contact between orbitosphenoid and frontal (vs. presence) [compare Figs. 3c, 4c and 5c]; (3) the ethmoid region being considerably shortened so that the orbitosphenoid is situated almost at the anterior tip of the skull (vs. orbitosphenoid separated from tip of skull by long mesethmoid crest) [compare Figs. 3c, 4c and 5c]; (4) absence of the exoccipital foramen (vs. presence) [compare Figs. 3b, 4b and 5b]; (5) the three branchiostegal rays being greatly elongated and curved dorsally ending close to the dorsal tip of the cleithrum (vs. not elongated and ending at midlevel of operculum) [compare Figs. 3a and f, 4a and f and 5a and f]; (6) the subopercle being extremely narrow and elongated reaching dorsally almost to dorsal margin of opercle (vs. shorter and ending well below dorsal margin of opercle) [compare Figs. 3a and f, 4a and f and 5a and f]; (7) the lateral process of vertebra 1 extending dorsally to cover the scaphium and most of centrum 1 laterally (vs. process short, knob-like, not extending dorsally) [compare Figs. 3e, 4e and 5e]; (8) absence of supraneural 2 (vs. presence) [compare Figs. 3e, 4e and 5e]; (9) the swimbladder capsule reaching posteriorly to anterior margin of 7th centrum (vs. only reaching to middle or posterior margin of 5th centrum) [compare Figs. 3g, 4g and 5g]; (10) the unique vertebral count of 29 abdominal + 19–20 caudal vertebrae [Figure 1c and supplementary Table 1]; (11) a unique count of 4 pelvic-fin rays (vs. more than 4 fin rays). It is further distinguished from all other cobitid taxa except the species of *Protocobitis*, *Paralepidocephalus translucens*, and the three subterranean species of *Pangio* from Kerala (*P. bhujia*, *P. juhuae*, *P. pathala*) by two troglomorphies: reduction or absence of eyes and of body pigment (vs. presence) (Fig. 1a, b,e, f). Another diagnostic feature is the presence of long nasal barbels (Fig. 1a, d,e, f), which is rare among cobitids, and only known from the tiny *Kottelatlimia katik*^15^and* Pangio bhujia*, *P. juhuae*, and *P. pathala*.

**Etymology**. The genus name is derived from the Garo word *gitchak*, meaning red, alluding to the striking red life colour of this loach. Treated here as a noun in the feminine gender.

***Gitchak nakana***, **new species**.

(Figs. 1 and 3)

LSID urn: lsid: zoobank.org: act: FBD7FA47-9FA9-4F98-967D-99633BCF0348.

Holotype.

ZSI FF11123, 20.8 mm SL; India: Assam: dug-out well in small village in Goalpara District, collected 30 March 2024, Leminard and Wimarithy K. Marak.

Paratypes.

ZSI FF11124, 2 specimens, 20.0–20.2 mm SL, same data as holotype. KUFOS.2025.FT.11.1, 20.8 mm SL, same data as holotype. ZSI_FF1125, 2 specimens, 14.0 mm SL, same locality as holotype, collected 01 March 2021, Wimarithy K. Marak. KUFOS.2025. FT.11.2-5, 4 specimens, 12.3–16.2 mm SL, same locality as holotype, collected 01 March 2021, Wimarithy K. Marak. KUFOS. 2025. FT.11.6, 1 specimen, 20.0 mm SL, same locality as holotype, collected 23 Feb 2025, Leminard K. Marak.

Additional non-type material.

KUFOS.2025.F.11.51, 3 specimens, 14.1–16.4 mm SL, same data as holotype.

**Diagnosis.** The same as that of genus.

**Etymology.** The specific name is derived from the Garo words *na·tok*, fish, and *kana*, blind, referring to the absence of eyes in this species, a noun in apposition.

**Distribution and habitat.** All 13 specimens of *Gitchak nakana* were collected from a single open dug-out well (Fig. 2) in a small village in the district of Goalpara, Assam. The well has a concrete ring above ground of 1 m in height and 1.5 m in diameter. From the top of this ring to the bottom, the well is 6.7 m deep and water depth was 3.6 m on 23 February 2025, but had risen to 5.6 m on 30 May 2025. Because this species has only been found in this single well and in low numbers, we decided not to disclose its precise locality, until its wider distribution and potential threats to its survival are better understood, see^16^. The well is situated in an area dominated by alluvial deposits according to the Geological Survey of India with “highly oxidised dark brown to reddish brown loamy sand” of Middle-Late Pleistocene age^17^.

**Description**. A miniature loach with a maximum size of 20.8 mm SL (Fig. 1). Body elongate, body depth 8.8–11.5 times in SL. Head 5.4–6.7 times in SL. Anterior head region with four pairs of long barbels (Fig. 1a, d,e, f): nasal and rostral barbels about 2/3 of head length (HL), maxillary barbel about 1/2 HL and mandibular barbel slightly shorter than 1/2 HL. Lower lip with a pair of very short mental barbels medial to mandibular barbels (Fig. 1f). In life, rostral, maxillary and mandibular barbels with large, wide blood vessels, occupying half of their diameter; nasal and minute mental barbels with only narrow blood vessels (Fig. 1f). Anterior naris at top of short tube arising from front of nasal barbel base; posterior naris a simple opening behind it. Gill opening not enlarged, ending dorsally above base of pectoral fin. Scales and body lateral-line canal absent.

Holotype (ZSI FF11123) with eight large eggs arranged in single series (Fig. 1a–d), eggs 1–1.2 mm wide and 1–1.5 mm long, two paratypes (ZSI FF11124, KUFOS.2025.FT.11.1) with five large eggs of similar size and developing oocytes. Swimbladder visible through transparent body wall (Fig. 1e). Body generally devoid of pigmentation, but melanophores present around nasal cavity, along a line separating left and right brain hemispheres, a line across epiphyseal brain area and along vertebral column above spinal cord (only visible in life) (Fig. 1a, e,f). Orbital area with tiny spot of pigment, covered by skin, on anterior side of head (Fig. 1e).

All fins transparent with segmented, but unbranched rays (Fig. 1a–c). Dorsal fin inserted between pelvic and anal fins, slightly posterior to middle of body (Fig. 1–c). Dorsal fin with 8 rays (*n* = 6), first of which tiny and covered by thick skin (Fig. 1a–c). Anal fin with 7 rays (*n* = 7), first ray tiny and embedded in thick skin, as in dorsal fin. Pectoral fin with 6 rays (*n* = 7). Pelvic fin with 4 rays (*n* = 7). Caudal fin with 6 + 7 (*n* = 1) or 7 + 7 (*n* = 6) main rays articulating with hypurals, plus 2–4 small dorsal and 1–4 small ventral rays in front of main rays. Vertebrae 29 + 19 (*n* = 5) or 29 + 20 (*n* = 2) = 48 or 49 (Fig. 1c).

### Osteology

*Neurocranium and infraorbital series* (Fig. 3a–c). Dorsal skull roof absent (Fig. 3a, c). Lateral line canals absent. Frontal restricted to side of cranium above anterior autosphenotic and pterosphenoid, well ossified along proximal margin but with very thin bone membrane distally towards dorsal midline (Fig. 3a–c). Parietal situated above posterior autosphenotic, also with better ossified proximal margin but thin irregular distal dorsally directed membranous bone. Supraoccipital U-shaped in dorsal view with large opening between left and right anterior sections (Fig. 3c). Ethmoid region short, with narrow, pointed, posterodorsally curved mesethmoid prong, fused at its base to triangular vomer (Fig. 3c). Orbitosphenoid situated far anteriorly, developed as a U-shaped, dorsally open band of bone with dorsolaterally directed wing above anterior tip of parasphenoid, approaching but not contacting anterior tip of frontal (Fig. 3c). Lateral margin of orbitosphenoid articulating with proximal base of lateral ethmoid. Lateral ethmoid developed into a bifid spine with short, blunt, lateral spinelet, and longer, more pointed, laterally curving, medial spine (Fig. 3c). Pterosphenoid small, with middle pierced by foramen and situated between ascending process of parasphenoid ventrally and frontal and autosphenotic dorsally, forming anterodorsal corner of very large trigeminofacial foramen (Fig. 3c). Autosphenotic an elongate bone of irregular shape contributing dorsal half of articular facet for hyomandibular, forming dorsoposterior margin of trigeminofacial foramen; also sutured to pterosphenoid anteriorly, frontal and parietal dorsally, pterotic posteriorly and prootic ventrally (Fig. 3b, c). Prootic large, forming ventral half of hyomandibular articular facet, and posterior and ventral margin of very large trigeminofacial foramen; also housing large sacculith in internal depression in posterior half (Fig. 3b, c). Pterotic capping side of posterior otic region, situated between autosphenotic anteriorly, prootic ventrally, epiotic posterodorsally, and exoccipital posteroventrally (Fig. 3b, c). Epiotic subcircular, small. Exoccipital large, entirely surrounding foramen magnum and forming lateral side wall of posterior otico-occipital region, without cypriniform exoccipital foramen (Fig. 3b). Parasphenoid broad and diamond shaped, tapering anteriorly and posteriorly, with ascending process at about midlength, contributing ventral and anteroventral margin to trigeminofacial foramen and with posterior tip of parasphenoid reaching only slightly beyond anterior margin of basioccipital; parasphenoid pierced by paired, laterally situated foramina in posterior half for internal carotid arteries (Fig. 3c). Basioccipital forming posterior base of skull and housing lagenolith in well-developed bulla; posterior aspect of basioccipital developed into articular facet for first vertebra and ventrally extended into pair of ventrally confluent pharyngeal processes, forming passage for dorsal aorta (Fig. 3b). Lacrimal, all other infraorbitals and supraorbital absent.

*Hyopalatine arch and hyoid* (Fig. 3d, f). Upper jaw bones, premaxilla and maxilla, well-developed; premaxilla with long ascending process for articulation with long kinethmoid and maxillary processes; maxilla deep, articulating anteriorly with small preautopalatine (Fig. 3d). Dentary long with broad coronoid process. Angulo-articular slender anteriorly but with wide articular surface for quadrate. Retroarticular forming posteroventral corner of lower jaw with long anterior process failing to reach posterior process from dentary. Hyomandibular with larger anterior and smaller posterior articular head and opercular articular head more ventrally situated at about half of hyomandibular length. Symplectic long. Quadrate large with prominent posteroventral process below symplectic, without quadrate metapterygoid fenestra (QMF, see^18) ^but straight cartilage-filled suture with metapterygoid (Fig. 3d). Metapterygoid not reaching hyomandibular, also without QMF, but two small circular openings (Fig. 3d); dorsoposterior corner of metapterygoid with small but sharp anterior process. Endopterygoid well developed with socket articulation for autopalatine. Ectopterygoid an hourglass shaped thin bone anterior to anterodorsal corner of quadrate. Hyoid bar with well-developed dorsal and ventral hypohyals, and elongate anterior and posterior ceratohyals (Fig. 3f). Interhyal rod-like articulating on dorsal socket at medial surface of posterior ceratohyal. Branchiostegal rays three, conspicuously long and thin, more than twice the length of entire hyoid bar, curving dorsally almost to dorsal most corner of opercle and tip of cleithrum. First branchiostegal articulating on medial face of anterior ceratohyal (on lateral face in illustrated specimen ZSI FF11124), second on lateral face of anterior and third on lateral face of posterior ceratohyal.

*Gill arches.* Comprising basihyal, three basibranchials, three hypobranchials, five ceratobranchials, four epibranchials and two pharyngobranchials. Basihyal prominent, with median constriction resulting in hour-glass shape in dorsal view. Basibranchial 1 largest, with wide anterior part articulating with hypohyal. 1 Basibranchial 2 also wider anteriorly, with tapering posterior half almost reaching hypobranchial 3. Basibranchial 3 a narrow, elongate rod in ventral midline behind basibranchial 2. Basibranchial 4 absent. Hypobranchials 1 and 2 well-developed, hypobranchial 3 small with transverse orientation. Ceratobranchials 1–4 developed as sequentially shorter rods. Ceratobranchial 5 larger than 4, with about 8 conical teeth: 5 functional, 3 replacement teeth.

*Shoulder girdle and pectoral fin.* Cleithrum large with wide middle section, tapering dorsally to a point, ventrally failing to meet antimere in midline. Supracleithrum a substantial elongate rod-like bone running along middle of cleithrum and connected to tiny, splint-like posttemporal on epiotic area of neurocranium. Scapula small articulating with uppermost pectoral-fin ray. Coracoid elongate and complexly shaped with dorsal mesocoracoid process along inner face of scapula, ventral process stopping short of ventral midline and not connected to coracoid on opposite side. Three pectoral radials, lower two fused at proximal end. Upper most with propterygium fused to medial tip of inner hemitrich.

*Weberian and post-Weberian vertebrae* (Fig. 3e, g). Centrum 1 with large dorsally curved process completely covering scaphium/claustrum complex laterally from view and closely abutting similar dorsally directed process of lateral process of centrum 2 (Fig. 3e). Neural arches 2 and 3 wide but shallow with shallow supraneural 3 articulating with both. Supraneural 2 absent (Fig. 3e). Neural spine 4 short, hardly extending posteriorly beyond level of centrum 3 posterior margin. Swimbladder capsule large, elongate, reaching posteriorly to anterior margin of vertebra 7 (Fig. 3g). Intermuscular bones absent.

*Dorsal*,* anal*,* pelvic and caudal fins* (Fig. 1c). Seven dorsal-fin pterygiophores, supporting seven serially associated fin rays and one supernumerary ray. Dorsal fin starting between neural spines of vertebrae 25 and 26, ending between vertebrae 28 and 29. Six anal-fin pterygiophores, supporting six serially associated fin rays and one supernumerary ray. Anal fin starting in front of hemal arch of vertebra 31 (first caudal) and ending in front of hemal arch of vertebra 35 (fifth caudal). Basipterygium of pelvic fin situated at level of vertebrae 21 and 22. Caudal fin skeleton consisting of two plates, lower plate comprising hypural and parhypural elements. Main caudal fin rays articulating with hypural plates, other small rays with epural and hemal spine of preural centrum 2.

### Molecular analyses

The final alignment length of data set 1 was 2,022 base pairs (cytb 1,113 and RAG1 909 base pairs) and the final alignment length for data set 2 (COI) was 639 base pairs. The result of the molecular divergence time estimates is shown in Fig. 5 (see Supplementary Fig. 1 for the uncollapsed tree) and the result of the ML analysis for data set 1 is shown in Supplementary Fig. 2. *Pangio* was not recovered as a monophyletic group in either analysis. Compared to the molecular divergence time estimate, the ML tree did not support monophyly of *Acanthopsoides* and further recovered the *Pangio shelfordii* group as the sister group to the remaining *Pangio* species groups plus the genera *Gitchak* and *Lepidocephalichthys* whereas in the BEAST analysis the *Pangio shelfordii* group was recovered as the sister group of the *Pangio anguillaris*- and *Pangio kuhlii*-*oblonga* groups, albeit with low bootstrap support (Fig. 6). The divergence time between *Gitchak* and its sister group, the *Pangio goaensis* group plus *Lepidocephalichthys*, was 33.4 my (confidence interval 21.4–45.5 my). The result of the ML analysis for data set 2 is shown in Supplementary Fig. 3. The two *Gitchak nakana* individuals had identical COI sequences. Minimum uncorrected *p*-distances between *Gitchak* and other subgroups of Cobitidae ranged from 16.7% to 20.7% (Supplementary Table 2).

## Discussion

(a) First phreatobitic fish from Northeast India.

Of the more than 37,500 currently recognized fish species^19^ only around 1% have conquered subterranean habitats and adapted to a life underground^7^. Approximately 70% of the 330 subterranean species listed in Proudlove^7^ occur in only five countries, with China and Brazil alone harbouring almost half of all subterranean fish diversity. With 21 known subterranean fishes, India is the country with the fourth greatest diversity. Its main centres of subterranean fish diversity are the north-eastern state of Meghalaya (6 spp.) and the south-western state of Kerala (13 spp.). While limestone caves are the dominant habitat of subterranean fishes in Meghalaya, their diversity in Kerala is restricted to lateritic aquifer-dwelling taxa, a globally highly unusual habitat for fishes^20^. Although such phreatobites have been found on all continents, they account for only a small fraction of subterranean fishes. The discovery of *Gitchak* from a well in Assam represents the first record of a phreatobitic cobitid from the Northeast of India, and also the first phreatobitic fish from there.

Unlike caves and their associated fauna, the phreatic zone is much more difficult to access and the discovery of phreatobitic fish species is usually serendipitous. This happens when wells, the only window into the phreatic realm, are emptied and cleaned and their hidden occupants literally come to light. Such chance encounters have led to the discovery of most phreatobitic taxa in the Western Ghats^9^ and probably world-wide, and are also the cause of the discovery of *Gitchak*. So far, this highly unusual cobitid loach has been found only in a single well, despite attempts to collect it from neighbouring wells. The entire geological setting of the type locality is so far unique for phreatobitic fish species. Situated in a highly geologically dynamic area, the alluvial deposits of the bed of a tributary of the Brahmaputra, this type of habitat is not expected to be stable for longer periods of geological time.

(b) Troglomorphies, subterranean life, miniaturization and highly derived morphological characters.

As its other subterranean cobitid and teleost relatives, *Gitchak* has a number of morphological characters associated with its phreatic life, so-called troglomorphies^2,4^: it lacks distinct body colouration and its translucent body appears blood-red, similar to the southern Indian phreatobitic species of the genus *Pangio*. Like many subterranean fishes it also lacks externally visible eyes and only has a tiny black spot in the orbital area of the head. Whether the lateral-line organ system shows any modifications associated with a subterranean life, such as proliferation of free neuromasts, is currently unknown. The only non-optic sense that may be considered enhanced, another feature of subterranean fishes, is the gustatory system, as its barbels, including its nasal barbels, are well-developed, comparatively long and densely covered in taste buds, although we do not know whether their number is comparatively greater than in other cobitids.

Another highly unusual character may also be related to the subterranean life of *Gitchak*. As exemplified by two mature females among our material, *Gitchak* has only few, but comparatively large eggs, while other cobitids usually have hundreds to thousands of eggs^21^. Few eggs of a comparatively large size have also been observed in the subterranean eel loach *Pangio bhujia* while its epigean relatives of *Pangio* may have more than one hundred eggs of only half their size^22^. As all eggs observed in *Gitchak* have roughly the same large size and stage, it is likely that all of them are spawned at the same time. This reliance on only few but large eggs would, after the resorption of the larger amount of yolk, most likely result in comparatively large offspring. And these larger youngh could potentially feed on similar food items as the adults. This may be important given the generally sparse availability of food in subterranean habitats^23^.

Phreatobitic subterranean fishes can be significantly smaller than their surface relatives^24^ and this is also true of *Gitchak*. With a maximum length of just around 20 mm SL, it is one of the smallest cobitids and qualifies as a miniature fish (sensu^25^). It is slightly smaller than the equally miniature subterranean *Pangio bhujia* and only slightly larger than the smallest cobitid species, the epigean *Kottelatlimia katik*.

Although *Gitchak* is not the only miniature subterranean cobitid, it has several highly unusual features in addition to the troglomorphies mentioned above: it is the only cobitid loach in which the skull roof is completely absent, so that left and right frontals and parietals are restricted to the dorsolateral part of the skull, creating a large dorsal skull opening covered only by skin. Due to its functional role as a protective structure of the brain, absence of a skull roof is rare among bony fishes, but has been reported from three other highly miniaturized cypriniforms: *Paedocypris*,* Danionella* and *Sundadanio*^26–29^, and other miniature teleosts^30–32^. In the cypriniform taxa, absence of the skull roof was shown to be the result of developmental truncation or progenesis, leading to tiny sexually mature taxa with larval features, a scenario that may also be applied to *Gitchak*. It also lacks the cobitid quadrato-metapterygoid foramen (QMF sensu^18^, which is otherwise commonly present in cobitids and to date only reported as absent from *Misgurnus*^13^. Absence in *Gitchak* of the cobitid typical QMF may also be attributed to progenesis, as the foramen is absent in earlier developmental stages of the cobitid *Cobitis*, only forming at sizes of 22 mm SL and above^18^. Often associated with highly developmentally truncated fishes are not only progenetic morphological structures, but also the presence of progressive, derived characters, which may represent evolutionary morphological novelties^26–28,33^. The greatly enlarged swimbladder and surrounding capsule in *Gitchak* may be such an example, as well as the elongated first lateral process, which covers the scaphium/claustrum complex laterally.

(c) Phylogenetic position among cobitid loaches and clade age.

Our result based on the analysis of cyt b and RAG1 sequence data, with the most comprehensive taxonomic coverage currently available, indicates that *Gitchak* forms a separate lineage and is part of a monophyletic group with a clade comprising the southern Indian species of *Pangio* and the genus *Lepidocephalichthys*, rendering the genus *Pangio* paraphyletic.

The results also show conflicting phylogenetic placements for the *Pangio shelfordii* group, either as the sister group to the remaining *Pangio* species groups (*P. goaensis*-, *anguillaris*- and *kuhlii*-*oblonga* groups) + *Gitchak* and *Lepidocephalichthys* in the ML analysis with a bootstrap support of 93% or as the sister group to the *P. anguillaris* and *P. kuhlii*-*oblonga* species groups in the molecular clock Bayesian analysis with a low posterior probability of 0.49. The limited data available from the cyt b and RAG1 sequences are clearly not sufficient to completely resolve the backbone of the tree and the phylogenetic interrelationships of the different groups of *Pangio* and the related genera *Gitchak* and *Lepidocephalichthys*. Additional phylogenomic and comprehensive osteological approaches may help us to better understand the evolution of Cobitidae.

Genetically, *Gitchak* is quite divergent from other Asian cobitids and shows an uncorrected *p*-distance in the barcoding COI gene of 16.7–23.3% to other South and Southeast Asian cobitid clades (Supplementary Table 2). Our time tree analysis indicates that *Gitchak* separated from its sister group at least 21.4–45.5 mya. This is unexpected as the habitat in which *Gitchak* was collected is located in the alluvial stratum of a tributary of the Brahmaputra and therefore is of Middle to Late Pleistocene age, i.e. under 1 my^17^. However, it is quite likely that this general kind of habitat, aquifers in alluvial deposits, has persisted in this area for a very long time. The neighbouring rocks, the Assam-Meghalaya Gneissic complex, are of Proterozoic age^17^, and could have been a potential source of sediments for the alluvial deposits created by the Brahmaputra and its tributaries in the area in which *Gitchak* occurs. An alternative explanation for the mismatch of the age of *Gitchak nakana* and that of its habitat is that its lineage included epigean ancestors that only entered the subterranean aquifer habitat in geologically more recent times.

(d) Wider significance of the discovery of *Gitchak*.

As the first phreatobitic fish species described from Northeast India, the discovery of *Gitchak* provides the first evidence that this landscape harbours a highly specialized subterranean phreatobitic fauna of the kind that previously has only been known from aquifers in the lateritic lowlands of the Western Ghats. Phreatobitic species are rare among subterranean fishes, and of the 272 valid species only around 23 come from groundwater aquifers^7^ and are usually found by chance when water is pumped or otherwise collected from aquifer-fed wells. It is especially these phreatobitic fish taxa that have proven difficult to place phylogenetically and while our phylogenetic analysis points to a close relationship with *Lepidocephalichthys* and southern Indian epigean and phreatobitic species of the genus *Pangio* this hypothesis needs further testing with more comprehensive molecular and morphological datasets. Ultimately arising from the discovery of *Gitchak* is the question of whether other subterranean taxa may be living in this previously unknown, unexplored and ultimately difficult to access habitat of aquifer-holding alluvial deposits in Northeast India. Studies are underway to address this question.

## Methods

### Ethical statement

No animal experiments were performed during our study. In accordance with European Union Directive 2010/63/EU (https://eur-lex.europa.eu/eli/dir/2010/63/oj/eng), all specimens were euthanized by Wimarithy K. Marak with an overdose of the anaesthetic clove oil prior to preservation.

### Morphology

13 specimens of this new loach species were collected from a well, euthanized with an anaesthetic overdose and subsequently preserved in 4% formaldehyde or 100% ethanol (EtOH). Length is provided as standard length (SL). Measurements reported in supplementary Table 1 were taken on the preserved specimens with an ocular micrometer fitted to a Zeiss Stemi 508 stereomicroscope with a precision of 0.1 mm.

One preserved specimen was cleared and double stained following the protocol of Taylor & van Dyke^34^. Specimens are deposited in the following collections: ZSI: Zoological Survey of India, Calcutta, India; KUFOS: Kerala University of Fisheries and Ocean Sciences, Kochi, India; ADBU-MF: Assam Don Bosco University, Museum of Fishes.

To obtain 3D whole-body information for vertebral and fin ray counts, seven preserved specimens including the holotype were µCT-scanned with the following parameters: 50-70 kV, 4–6 W, 4.9–8.8 μm voxel size, 1–3 s. exposure time, 801–1401 projections, 1–2 frames per projection and no filter. For detailed information on the head skeleton and Weberian apparatus, two paratypes (ZSI FF11124, 20.2 mm SL; KUFOS.2025.FT.11.6, 20 mm SL) was also scanned with the following parameters: 50 kV, 4 W, 2.6 μm voxel size, 2.5 s. exposure time, 3201 projections, 5 frames per projection and no filter. For comparison with other Indian cobitids, one specimen of *Lepidocephalichthys guntea* (ADBU-MF 5597, 56.6 mm SL, Fig. 4) and one of *Pangio pangia* (ADBU-MF 5282, Fig. 5) were also scanned: *L. guntea* at 70 kV, 6 W, 4.9 μm voxel size, 1.2 s exposure time, 4501 projections, 4 frames per projection and no filter and *P. pangia* 60 kV, 5 W, 3.8 μm voxel size, 2.5 s exposure time, 4001 projections, 3 frames per projection and no filter. Images in Figs. 2, 3 and 4, were generated with the software Amira^®^ after segmentation in Dragonfly^®^.

### Molecular

Laboratory procedures including DNA extraction from tissues preserved in 100% EtOH, PCR amplification and Sanger sequencing of the new loach followed protocols by Ali et al.^35^ and Sidharthan et al.^36^ using the following PCR primers: recombination activation protein 1 (RAG1), R12533F and R14078R^37^; cytochrome b (cyt b), DonGluF2533 DonThrR2535^38^; cytochrome c oxidase subunit 1 (COI), FishF1 and FishR2^39^ and those of the remaining samples followed^40^ using the same primers as above. Raw reads were edited and assembled into contigs using Geneious Prime v2022.0.2 (https://www.geneious.com) and individual consensus sequences together with sequences available at GenBank (see below) were aligned using MAFFT v7.017^41^, as implemented in Geneious Prime.

To place the new loach into a larger phylogenetic context, we assembled a comprehensive data set (data set 1) for which a broad cobitid taxon sample is available. It consists of the mitochondrial cyt b and the nuclear RAG1 genes, genetic markers that were used in previous molecular studies on Cobitidae^42,43^. The data set includes a total of 178 specimens, with one representative of the Botiidae as outgroup, four representatives of the Nemacheilidae and 173 representatives of the Cobitidae. Four individuals (two of *Gitchak nakana*, one each of *Pangio* sp. South Kerala and *Pangio* sp. Kerala) were sequenced specifically for this study, and an additional 169 sequences were downloaded from GenBank. To assess the genetic diversity among close relatives of the new loach, we assembled a comprehensive DNA barcode data set (data set 2) based on COI. The data set includes a total of 549 individuals, including eight representatives of the northern Cobitidae clade *sensu* Šlechtová et al.^42^ used as outgroups. Eleven individuals were sequenced specifically for this study including seven representatives of the *Pangio shelfordii* group, and another 533 sequences were downloaded from GenBank. All newly generated sequences have been deposited in Genbank (see data availability statement).

Maximum likelihood analyses with 1,000 ultrafast bootstrap replicates were conducted for both alignments using IQ-TREE v2.3.6^44^ and an initial partition scheme by gene and codon position using the model option “-m MFP+MERGE” that first looks for the best-fit partitioning scheme, immediately followed by tree reconstruction using the best partitioning scheme found. A divergence time analysis for alignment 1 was carried out in BEAST v2.6.6^45^ using a secondary calibration for the Cobitidae crown group. Previous molecular divergence time analyses found the following age estimates for this node: Šlechtová et al.^42^: 50.48 mya, Betancur-R et al.^46^: 39.83 million years (my), Rabosky et al.^47^: 53.80 my, Hughes et al.^48^: 54.21 my (confidence interval 39.54–70.53 my), Šlechtová et al.^49^: 52.43 my, Šlechtová et al.^50^: 35.71 my (confidence interval 28.44–45.47 my). For our secondary calibration we used the age estimate by Hughes et al.^41^. For the BEAST analysis we used five partitions as suggested by IQ-TREE and we unlinked the site models and linked all clock and tree models. For each partition we used the GTR model with four gamma rate categories and all other parameters set to estimation. We used a relaxed molecular clock with a log-normal distribution as our clock prior and set the tree prior to the Yule model. For all the remaining priors we used the default settings. The tree was calibrated by applying a normal distribution to the crown group age of Cobitidae (see above, offset = 54.21 and sigma = 8). We ran the Markov chain Monte Carlo (MCMC) for 5 × 10^7^ generations with a sample frequency of 2,000 and we used a conservative burn-in of 50%. Chain convergence and effective sample size (ESS) to ensure adequate mixing of the MCMC were assessed using Tracer v1.5 and TreeAnnotator^51^ was used to summarize the information from the posterior of the post burn‐in samples in a maximum clade credibility (MCC) tree. The posterior probability for each clade and the mean and 95% posterior density estimates of the corresponding divergence times were visualized in FigTree v1.4.4 (http://tree.bio.ed.ac.uk/software/Figtree/). Genetic distances (uncorrected *p*-distances) within, and between, selected species or clades for COI were calculated in PAUP* v4.0a147^52^.

## Supplementary Information

Below is the link to the electronic supplementary material.

Supplementary Material 1

Supplementary Material 2

Supplementary Material 3

## Data Availability

All newly generated sequences analysed during the current study are available in the Genbank repository (https://www.ncbi.nlm.nih.gov/genbank/) under accession numbers PX277237-PX277247 for COI, PX289534-PX289537 for cyt b and PX289538-PX289541 for RAG1 and the data sets generated and analysed for our study are available as supplementary information files.
