# Supplementary material for: A miniature, subterranean, blind cobitid loach, Gitchak nakana, new genus and species, is the first groundwater-dwelling fish from Northeast India
Source: Sci Rep. 2026 Feb 26;16:7746. doi: 10.1038/s41598-026-40425-6 (PMC12949253; doi:10.1038/s41598-026-40425-6)
Supplement: Supplementary file 3 — Supplementary Material 3 [file 41598_2026_40425_MOESM3_ESM.pdf]

Supplementary material for

A miniature, subterranean, blind cobitid loach, *Gitchak nakana*, new genus and species, is the first groundwater-dwelling fish from Northeast India

Ralf Britz[1], Wimarithy K. Marak[2], Kangjam Velentina[2], Yumnam Lokeshwor[3], Rajeev Raghavan[4], Amanda K. Pinion[1], and Lukas Rüber[5,6]

[1]Senckenberg Naturhistorische Sammlungen Dresden, Germany. E-mail: ralf.britz@senckenberg.de
[2]Assam Don Bosco University, Guwahati, Assam, India
[3]Dhanamanjuri University, Imphal, Manipur, India
[4]Department of Fisheries Resource Management, Kerala University of Fisheries and Ocean Studies (KUFOS), Kochi, India
[5]Naturhistorisches Museum Bern, Switzerland
[6]Aquatic Ecology and Evolution, Institute of Ecology and Evolution, University of Bern, Bern, Switzerland

Supplementary table 1. Measurements and counts of holotype and select paratypes of *Gitchak nakana*.

	ZSI FF11123	ZSI FF11124	ZSI FF11124	KUFOS 2025.FT.11.1	ZSI FF11125	KUFOS 2025.FT.11.2-5	ZSI FF11125	KUFOS 2025.FT.11.2-5	KUFOS 2025.FT.11.2-5	KUFOS 2025.FT.11.2-5
measurements										
SL	20.8	20	20.2	20.8	14.0	16.2	14.0	12.3	12.5	15.5
in % SL										
HL	15.4	16	14.9	15.4	17.9	17.3	18.6	19.5	19.2	18.7
Prepelvic length	54.3	52.5	49.5	48.1	45.7	45.1	53.6	52.0	56.0	50.3
Predorsal length	63.5	63.5	59.4	61.5	64.3	54.3	62.9	61.0	64.0	59.4
Preanal length	70.2	68.5	66.8	69.7	63.9	63.6	69.3	67.5	71.2	65.8
Caudal peduncle length	30.3	32.5	34.2	32.7	29.3	34.0	25	32.5	28.8	30.3
Body depth at pectoral fin	10.6	10.0	9.9	9.1	12.1	10.5	11,4	13.0	12.0	11.6
Body depth at dorsal fin	11.5	11.0	8.9	9.6	12.1	10.5	11,4	11.4	10.4	9.7
Caudal peduncle depth	8.2	9,5	6.9	7.2	10.0	11.7	9.3	10.6	8.8	9
Body width at dorsal fin	6.3	5,5	5.4	5.8	5.7	6.8	6.4	6.5	5.6	6.5
counts										
vertebrae										
abdominal	29	29	29	29		29				29
caudal	20	19	19	19		19				19
total	49	48	48	48		48				48
fin rays										
dorsal	8	8	8	8		8				8
anal	7	7	7	7		7				7
caudal	7+7	7+7	7+7	7+7		6+7				7+7
pectoral	6	6	6	6		6				6
pelvic	4	4	4	4		4				4

Supplementary table 2. Compilation of vertebral numbers among members of the family Cobitidae.

species	abdominal	caudal	total vertebrae	source
Gitchak nakana holotype	29	20	49	this study, CT, ZSI FF11123
Gitchak nakana paratype	29	19	48	this study, CT, ZSI FF11124
Gitchak nakana paratype	29	19	48	this study, CT, ZSI FF11124
Gitchak nakana paratype	29	19	48	this study, CT, KUFOS.2025.FT.11.1
Gitchak nakana paratype	29	19	48	this study, c&s, KUFOS.2025.FT.11.2-5
Gitchak nakana paratype	29	19	48	this study, CT, KUFOS.2025.FT.11.2-5
Gitchak nakana paratype	29	20	49	this study, CT, KUFOS.2025.FT.11.6
Acantopsis choirorhynchos	28–29	13–14	42	Roberts 1989
Acantopsis lachnostoma	29	15	44	Roberts 1989
Acanthopsoides gracilis	27–28	12–14	40–42	Roberts 1989
Cobitis elongatoides	31	14	45	this study, CT, MTD 28580–28644
Kottelatlimia pristes	23–24	9–10	33–34	Roberts 1989
Kottelatlimia hipporhynchos	23–24	10–12	34–36	Kottelat & Lim 1992
Kottelatlimia katik	20	8–9	28–29	Kottelat & Lim 1992
Lepdiocephalichthys berdmorei	26–28	10–13	37–39	Kottelat & Lim 1992
Lepidocephalichthys cf. hasselti	24–25	11–13	34–37	Kottelat & Lim 1992
Lepidocephalichthys furcatus	22–24	10–11	32–34	Kottelat & Lim 1992
Lepidocephalichthys guntea	25–26	11	36–37	Kottelat & Lim 1992
Lepidocephalichthys guntea	24–25	11	35–36	Roberts 1989
Lepidocephalichthys hasselti	22–25	10–12	33–37	Kottelat & Lim 1992
Lepidocephalichthys hasselti	24–25	11–12	35–36	Roberts 1989
Lepidocephalichthys jonklaasi	25–26	10	35–36	Kottelat & Lim 1992
Lepidocephalichthys lorentzi	25–26	11–12	36–38	Roberts 1989
Lepidocephalichthys lorentzi	25–26	10–11	36	Kottelat & Lim 1992
Lepidocephalichthys micropogon	23–24	11–13	35–36	Kottelat & Lim 1992
Lepidocephalichthys thermalis	24–25	10–11	35–36	Kottelat & Lim 1992
Lepidocephalichthys tomaculum	24–26	10–11	34–36	Kottelat & Lim 1992
Lepidocephalus macrochir	28	15	43	Roberts 1989
Lepidocephalus spectrum	28	14–15	42–43	Roberts 1989
Misgurnus fossilis	35	17	52	this study
Pangio agma	35	13	48	Kottelat & Lim 1993
Pangio alcoides	39–40	12–16	51–55	Kottelat & Lim 1993
Pangio alternans	32–34	13–15	45–48	Kottelat & Lim 1993
Pangio ammophila	33	15	48	Britz et al. 2012
Pangio anguillaris	50–52	19–20	69–71	Kottelat & Lim 1993, Roberts 1989
Pangio apoda	38–39	12–14	51–53	Britz & Maclaine 2007
Pangio atactos	34–36	14–16	49–51	Tan & Kottelat 2009
Pangio bhujia	38–39	23–24	62–63	Anoop et al. 2019
Pangio bitaimac	46–49	14–17	61–64	Tan & Kottelat 2009
Pangio cf pangia Myitkina	40	14–15	54–55	Roberts 1989
Pangio cuneovirgata	35–36	14–15	49–51	Kottelat & Lim 1993
Pangio doriae	46–49	15–18	62–67	Kottelat & Lim 1993
Pangio elongata	44–46	13–15	57–62	Britz & Maclaine 2007
Pangio filinaris	33–36	12–14	45–49	Kottelat & Lim 1993
Pangio fusca	41–44	11–15	52–58	Britz & Maclaine 2007
Pangio incognito	32	13–14	45–46	Kottelat & Lim 1993
Pangio juhuae	?	?	63	Sreenath et al. 2025, counted from fig. 5
Pangio kuhlii	34–37	12–15	47–50	Kottelat & Lim 1993
Pangio lidi	41–42	17–18	59	Hadiaty & Kottelat 2009
Pangio longimana	32–33	13–14	46–47	Britz & Kottelat 2010
Pangio lumbriciformis	40–41	13–15	54–56	Britz & Maclaine 2007
Pangio malayana	35–37	13–14	49–51	Kottelat & Lim 1993
Pangio muraeniformis	33–35	14–16	48–50	Roberts 1989
Pangio myersi	35	13	48	Roberts 1989
Pangio oblonga	33–35	12	45–47	Roberts 1989
Pangio oblonga	33	12–14	45–47	Kottelat & Lim 1993
Pangio pangia Myanmar	34–39	11–14	47–52	Britz & Maclaine 2007
Pangio pathala	40	27	67	Sundar et al. 2022
Pangio piperata	31–35	13–16	46–49	Kottelat & Lim 1993
Pangio pulla	46–48	15–16	61–62	Kottelat & Lim 1993
Pangio semicincta	33–37	12–15	46–51	Roberts 1989
Pangio shelfordii	32–36	14–16	46–51	Kottelat & Lim 1993, Roberst 1989
Pangio signicauda	40–42	13–15	54–56	Britz & Maclaine 2007
Pangio superba	35–36	15–16	50–52	Roberts 1989
Protocobitis longibarba	31	16	47	Quin et al. 2025, counted from fig. 1
Protocobitis longicostatus	31	16	47	Zhou et al. 2024, counted from fig. 1
Protocobitis polylepis	29	14	43	Zhou et al. 2024, counted from fig. 1
Protocobitis typhlops	32	14	46	Zhou et al. 2024, counted from fig. 1
Sabanejewia aurata	28	16	44	this study, CT, MTD11892
Theriodes sandakanensis	23–25	11–13	35–36	Roberts 1989
Theriodes sandakanensis	25–26	10–11	35–36	Kottelat & Lim 1992

Supplementary table 3. Range of pairwise genetic distances (*p*- distances) in the DNA barcoding gene COI between *Gitchak nakana* and select clades of Cobitidae.

Gitchak nakana	*Gitchak nakana* (n=2)	0.00
	Acanthopsoides gracilentus (n=1)	0.190
	Kottelatlimia pristes (n=1)	0.204
	Lepidocephalus macrochir (n=1)	0.221
	Canthophrys gongota (n=37)	0.207–0.222
	Acantopsis (n=124)	0.181–0.209
	Neoeucirrhichtys maydelli (n=4)	0.199–0.209
	Pangio goaensis group (n=5)	0.195–0.209
	Pangio bhujia group (n=5)	0.185–0.195
	Pangio shelfordii group (n=7)	0.167–0.206
	Pangio anguillaris group (n=3)	0.185–0.195
	Pangio kuhlii–oblonga group (n=35)	0.167–0.191
	Lepidocephalichthys (n=309)	0.168–0.233

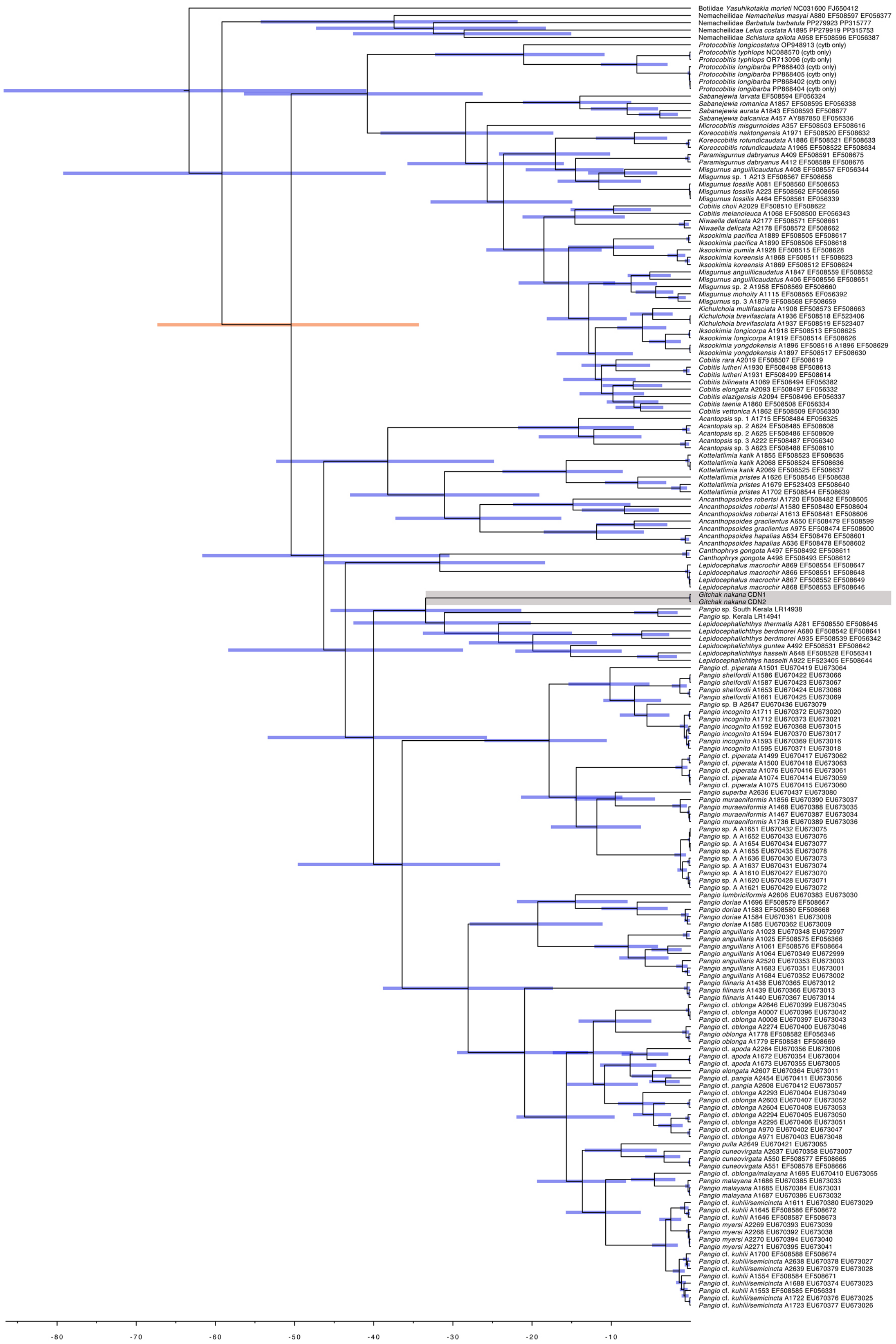

Supplementary Figure 1. Molecular time tree from the BEAST analysis of the combined cytb and rag1 data set of Cobitidae. The secondary calibration interval obtained from the literature used to calibrate the tree is highlighted in red. *Gitchak nakana* is highlighted in grey.

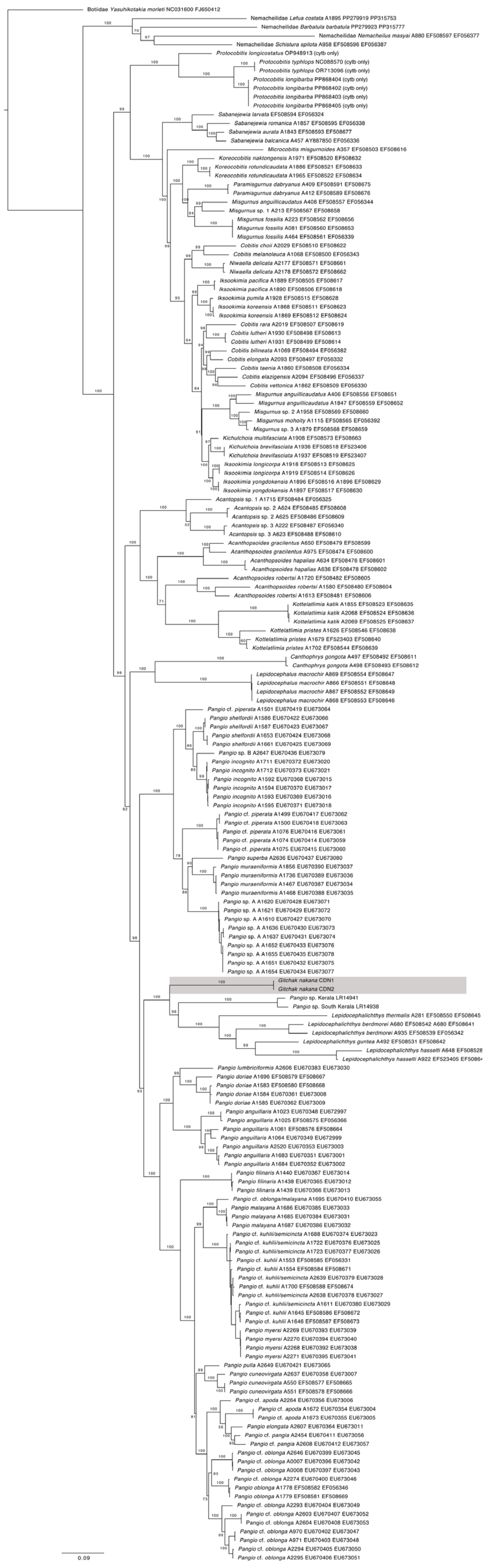
Botiidae Yasuhikotakia morleti NC031600 FJ650412
Nemacheilidae Lefua costata A1895 PP279919 PP315753
Nemacheilidae Barbatula barbatula PP279923 PP315777
Nemacheilidae Nemacheilus masyai A880 EF508597 EF056377
Nemacheilidae Schistura spilota A958 EF508596 EF056387
Protocobitis longicostatus OP948913 (cytb only)
Protocobitis typhlops NC088570 (cytb only)
Protocobitis typhlops OR713096 (cytb only)
Protocobitis longibarba PP868404 (cytb only)
Protocobitis longibarba PP868402 (cytb only)
Protocobitis longibarba PP868403 (cytb only)
Protocobitis longibarba PP868405 (cytb only)
Sabanejewia larvata EF508594 EF056324
Sabanejewia romanica A1857 EF508595 EF056338
Sabanejewia aurata A1843 EF508593 EF508677
Sabanejewia balcanica A457 AY887850 EF056336
Microcobitis misgurnoides A357 EF508503 EF508616
Koreocobitis naktongensis A1971 EF508520 EF508632
Koreocobitis rotundicaudata A1886 EF508521 EF508633
Koreocobitis rotundicaudata A1965 EF508522 EF508634
Paramisgurnus dabryanus A409 EF508591 EF508675
Paramisgurnus dabryanus A412 EF508589 EF508676
Misgurnus anguillicaudatus A408 EF508557 EF056344
Misgurnus sp. 1 A213 EF508567 EF508658
Misgurnus fossilis A223 EF508562 EF508656
Misgurnus fossilis A081 EF508560 EF508653
Misgurnus fossilis A464 EF508561 EF056339
Cobitis choii A2029 EF508510 EF508622
Cobitis melanoleuca A1068 EF508500 EF056343
Niwaella delicata A2177 EF508571 EF508661
Niwaella delicata A2178 EF508572 EF508662
Iksookimia pacifica A1889 EF508505 EF508617
Iksookimia pacifica A1890 EF508506 EF508618
Iksookimia pumila A1928 EF508515 EF508628
Iksookimia koreensis A1868 EF508511 EF508623
Iksookimia koreensis A1869 EF508512 EF508624
Cobitis rara A2019 EF508507 EF508619
Cobitis lutheri A1930 EF508498 EF508613
Cobitis lutheri A1931 EF508499 EF508614
Cobitis bilineata A1069 EF508494 EF056382
Cobitis elongata A2093 EF508497 EF056332
Cobitis taenia A1860 EF508508 EF056334
Cobitis elazigensis A2094 EF508496 EF056337
Cobitis vettonica A1862 EF508509 EF056330
Misgurnus anguillicaudatus A406 EF508556 EF508651
Misgurnus anguillicaudatus A1847 EF508559 EF508652
Misgurnus sp. 2 A1958 EF508569 EF508660
Misgurnus mohoity A1115 EF508565 EF056392
Misgurnus sp. 3 A1879 EF508568 EF508659
Kichulchoia multifasciata A1908 EF508573 EF508663
Kichulchoia brevifasciata A1936 EF508518 EF523406
Kichulchoia brevifasciata A1937 EF508519 EF523407
Iksookimia longicorpa A1918 EF508513 EF508625
Iksookimia longicorpa A1919 EF508514 EF508626
Iksookimia yongdokensis A1896 EF508516 A1896 EF508629
Iksookimia yongdokensis A1897 EF508517 EF508630
Acantopsis sp. 1 A1715 EF508484 EF056325
Acantopsis sp. 2 A624 EF508485 EF508608
Acantopsis sp. 2 A625 EF508486 EF508609
Acantopsis sp. 3 A222 EF508487 EF056340
Acantopsis sp. 3 A623 EF508488 EF508610
Acanthopsoides gracilentus A650 EF508479 EF508599
Acanthopsoides gracilentus A975 EF508474 EF508600
Acanthopsoides hapalias A634 EF508476 EF508601
Acanthopsoides hapalias A636 EF508478 EF508602
Acanthopsoides robertsi A1720 EF508482 EF508605
Acanthopsoides robertsi A1580 EF508480 EF508604
Acanthopsoides robertsi A1613 EF508481 EF508606
Kottelatlimia katik A1855 EF508523 EF508635
Kottelatlimia katik A2068 EF508524 EF508636
Kottelatlimia katik A2069 EF508525 EF508637
Kottelatlimia pristes A1626 EF508546 EF508638
Kottelatlimia pristes A1679 EF523403 EF508640
Kottelatlimia pristes A1702 EF508544 EF508639
Canthophrys gongota A497 EF508492 EF508611
Canthophrys gongota A498 EF508493 EF508612
Lepidocephalus macrochir A869 EF508554 EF508647
Lepidocephalus macrochir A866 EF508551 EF508648
Lepidocephalus macrochir A867 EF508552 EF508649
Lepidocephalus macrochir A868 EF508553 EF508646
Pangio cf. piperata A1501 EU670419 EU673064
Pangio shelfordii A1586 EU670422 EU673066
Pangio shelfordii A1587 EU670423 EU673067
Pangio shelfordii A1653 EU670424 EU673068
Pangio shelfordii A1661 EU670425 EU673069
Pangio sp. B A2647 EU670436 EU673079
Pangio incognito A1711 EU670372 EU673020
Pangio incognito A1712 EU670373 EU673021
Pangio incognito A1592 EU670368 EU673015
Pangio incognito A1594 EU670370 EU673017
Pangio incognito A1593 EU670369 EU673016
Pangio incognito A1595 EU670371 EU673018
Pangio cf. piperata A1499 EU670417 EU673062
Pangio cf. piperata A1500 EU670418 EU673063
Pangio cf. piperata A1076 EU670416 EU673061
Pangio cf. piperata A1074 EU670414 EU673059
Pangio cf. piperata A1075 EU670415 EU673060
Pangio superba A2636 EU670437 EU673080
Pangio muraeniformis A1856 EU670390 EU673037
Pangio muraeniformis A1736 EU670389 EU673036
Pangio muraeniformis A1467 EU670387 EU673034
Pangio muraeniformis A1468 EU670388 EU673035
Pangio sp. A A1620 EU670428 EU673071
Pangio sp. A A1621 EU670429 EU673072
Pangio sp. A A1610 EU670427 EU673070
Pangio sp. A A1636 EU670430 EU673073
Pangio sp. A A1637 EU670431 EU673074
Pangio sp. A A1652 EU670433 EU673076
Pangio sp. A A1655 EU670435 EU673078
Pangio sp. A A1651 EU670432 EU673075
Pangio sp. A A1654 EU670434 EU673077
Gitchak nakana CDN1
Gitchak nakana CDN2
Pangio sp. Kerala LR14941
Pangio sp. South Kerala LR14938
Lepidocephalichthys thermalis A281 EF508550 EF508645
Lepidocephalichthys berdmorei A680 EF508542 A680 EF508641
Lepidocephalichthys berdmorei A935 EF508539 EF056342
Lepidocephalichthys guntea A492 EF508531 EF508642
Lepidocephalichthys hasselti A648 EF50852
Lepidocephalichthys hasselti A922 EF523405 EF5086
Pangio lumbriciformis A2606 EU670383 EU673030
Pangio doriae A1696 EF508579 EF508667
Pangio doriae A1583 EF508580 EF508668
Pangio doriae A1584 EU670361 EU673008
Pangio doriae A1585 EU670362 EU673009
Pangio anguillaris A1023 EU670348 EU672997
Pangio anguillaris A1025 EF508575 EF056366
Pangio anguillaris A1061 EF508576 EF508664
Pangio anguillaris A1064 EU670349 EU672999
Pangio anguillaris A2520 EU670353 EU673003
Pangio anguillaris A1683 EU670351 EU673001
Pangio anguillaris A1684 EU670352 EU673002
Pangio filinaris A1440 EU670367 EU673014
Pangio filinaris A1438 EU670365 EU673012
Pangio filinaris A1439 EU670366 EU673013
Pangio cf. oblonga/malayana A1695 EU670410 EU673055
Pangio malayana A1686 EU670385 EU673033
Pangio malayana A1685 EU670384 EU673031
Pangio malayana A1687 EU670386 EU673032
Pangio cf. kuhlii/semicincta A1688 EU670374 EU673023
Pangio cf. kuhlii/semicincta A1722 EU670376 EU673025
Pangio cf. kuhlii/semicincta A1723 EU670377 EU673026
Pangio cf. kuhlii A1553 EF508585 EF056331
Pangio cf. kuhlii A1554 EF508584 EF508671
Pangio cf. kuhlii/semicincta A2639 EU670379 EU673028
Pangio cf. kuhlii A1700 EF508588 EF508674
Pangio cf. kuhlii/semicincta A2638 EU670378 EU673027
Pangio cf. kuhlii/semicincta A1611 EU670380 EU673029
Pangio cf. kuhlii A1645 EF508586 EF508672
Pangio cf. kuhlii A1646 EF508587 EF508673
Pangio myersi A2269 EU670393 EU673039
Pangio myersi A2270 EU670394 EU673040
Pangio myersi A2268 EU670392 EU673038
Pangio myersi A2271 EU670395 EU673041
Pangio pulla A2649 EU670421 EU673065
Pangio cuneovirgata A2637 EU670358 EU673007
Pangio cuneovirgata A550 EF508577 EF508665
Pangio cuneovirgata A551 EF508578 EF508666
Pangio cf. apoda A2264 EU670356 EU673006
Pangio cf. apoda A1672 EU670354 EU673004
Pangio cf. apoda A1673 EU670355 EU673005
Pangio elongata A2607 EU670364 EU673011
Pangio cf. pangia A2454 EU670411 EU673056
Pangio cf. pangia A2608 EU670412 EU673057
Pangio cf. oblonga A2646 EU670399 EU673045
Pangio cf. oblonga A0007 EU670396 EU673042
Pangio cf. oblonga A0008 EU670397 EU673043
Pangio cf. oblonga A2274 EU670400 EU673046
Pangio oblonga A1778 EF508582 EF056346
Pangio oblonga A1779 EF508581 EF508669
Pangio cf. oblonga A2293 EU670404 EU673049
Pangio cf. oblonga A2603 EU670407 EU673052
Pangio cf. oblonga A2604 EU670408 EU673053
Pangio cf. oblonga A970 EU670402 EU673047
Pangio cf. oblonga A971 EU670403 EU673048
Pangio cf. oblonga A2294 EU670405 EU673050
Pangio cf. oblonga A2295 EU670406 EU673051
0.09

Supplementary Figure 2. Rooted phylogram of maximum likelihood analysis of combined cytb and rag1 data set of the Cobitidae using selected Nemacheilidae taxa as outgroups. Bootstrap support values are shown above or below selected branches. *Gitchak nakana* is highlighted in grey.

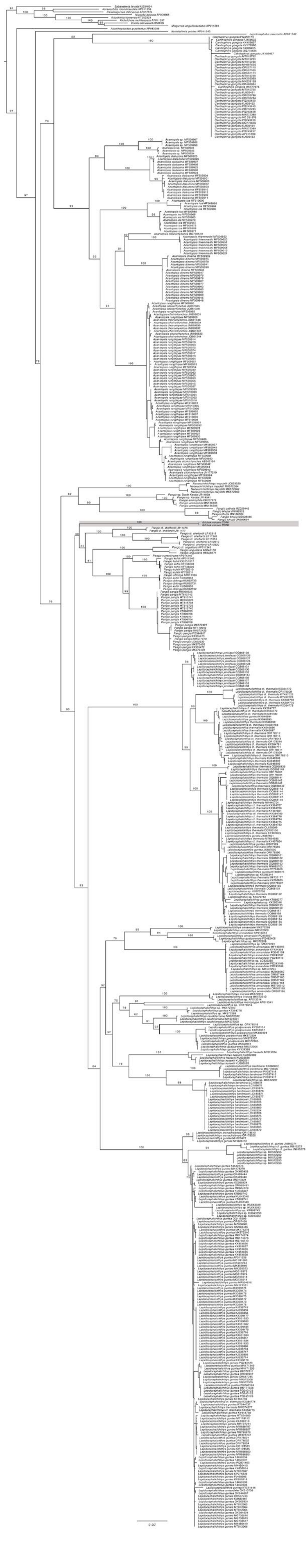

Supplementary Figure 3. Rooted phylogram of maximum likelihood analysis of barcoding portion of COI gene for southern Cobitidae clade using selected taxa from northern Cobitidae clade as outgroups. Bootstrap support values are shown above selected branches. *Gitchak nakana* is highlighted in grey.
